# Fires, Smoke Exposure, and Public Health: An Integrative Framework to Maximize Health Benefits From Peatland Restoration

**DOI:** 10.1029/2019GH000191

**Published:** 2019-07-24

**Authors:** Miriam E. Marlier, Tianjia Liu, Karen Yu, Jonathan J. Buonocore, Shannon N. Koplitz, Ruth S. DeFries, Loretta J. Mickley, Daniel J. Jacob, Joel Schwartz, Budi S. Wardhana, Samuel S. Myers

**Affiliations:** ^1^ The RAND Corporation Santa Monica CA USA; ^2^ Department of Ecology, Evolution, and Environmental Biology Columbia University New York NY USA; ^3^ Department of Earth and Planetary Sciences Harvard University Cambridge MA USA; ^4^ School of Engineering and Applied Sciences Harvard University Cambridge MA USA; ^5^ Center for Climate, Health, and the Global Environment, Harvard T.H. Chan School of Public Health Harvard University Boston MA USA; ^6^ Harvard T.H. Chan School of Public Health Harvard University Boston MA USA; ^7^ Badan Restorasi Gambut Jakarta Indonesia; ^8^ Harvard University Center for the Environment Harvard University Cambridge MA USA

**Keywords:** planetary health, fires, biomass burning, land use, smoke, peatland restoration

## Abstract

Emissions of particulate matter from fires associated with land management practices in Indonesia contribute to regional air pollution and mortality. We assess the public health benefits in Indonesia, Malaysia, and Singapore from policies to reduce fires by integrating information on fire emissions, atmospheric transport patterns, and population exposure to fine particulate matter (PM_2.5_). We use adjoint sensitivities to relate fire emissions to PM_2.5_ for a range of meteorological conditions and find that a Business‐As‐Usual scenario of land use change leads, on average, to 36,000 excess deaths per year into the foreseeable future (the next several decades) across the region. These deaths are largely preventable with fire reduction strategies, such as blocking fires in peatlands, industrial concessions, or protected areas, which reduce the health burden by 66, 45, and 14%, respectively. The effectiveness of these different strategies in mitigating human health impacts depends on the location of fires relative to the population distribution. For example, protecting peatlands through eliminating all fires on such lands would prevent on average 24,000 excess deaths per year into the foreseeable future across the region because, in addition to storing large amounts of fuel, many peatlands are located directly upwind of densely populated areas. We also demonstrate how this framework can be used to prioritize restoration locations for the Indonesian Peatland Restoration Agency based on their ability to reduce pollution exposure and health burden. This scientific framework is publicly available through an online decision support tool that allows stakeholders to readily determine the public health benefits of different land management strategies.

## Key Points

- Current trends in Indonesia's fire activity would lead to 37,000 excess deaths per year across the region from air pollution exposure
- Restoring peatlands could reduce fire‐related mortality by nearly 70%
- Our integrative scientific tool can be used to support land management strategies most beneficial for public health

## 1 Introduction

Over the past several decades, biomass burning in Indonesia has become a substantial contributor to global fire emissions (van der Werf et al., 2017) and health impacts due to regional air pollution (Johnston et al., 2012; Koplitz et al., 2016; Marlier et al., 2013). Most recently, the severe haze that blanketed equatorial Asia during 2015 was a stark reminder of the climate change and public health consequences associated with land management activities in Indonesia. The fires, which peaked during September and October of 2015, released CO_2_ emissions comparable to Japan or India's annual fossil fuel emissions (Field et al., 2016), exposed more than 69 million people to unhealthy air (Crippa et al., 2016), and cost more than $16 billion USD, without considering long‐term health or ecosystem impacts (World Bank Group, 2016).

In this study, we present a novel approach that integrates information on the drivers of fire emissions in Indonesia (Marlier, DeFries, Kim, Gaveau, et al., 2015; Marlier, DeFries, Kim, Koplitz, et al., 2015; Marlier, DeFries, Pennington, Nelson, et al., 2015), the transport of smoke to downwind regional population centers (Kim et al., 2015), and the resulting population exposure to air pollution (Koplitz et al., 2016) in order to quantify the health impacts of different land management scenarios. We apply this framework to prioritize locations for peatland restoration sites to reduce population exposure to fire emissions in Indonesia, Malaysia, and Singapore and to develop an online decision support tool to evaluate the efficacy of other potential policy scenarios. This integrative framework is applicable to fire‐prone regions around the world to develop strategies that reduce negative health outcomes associated with biomass burning.

**Figure 1:**
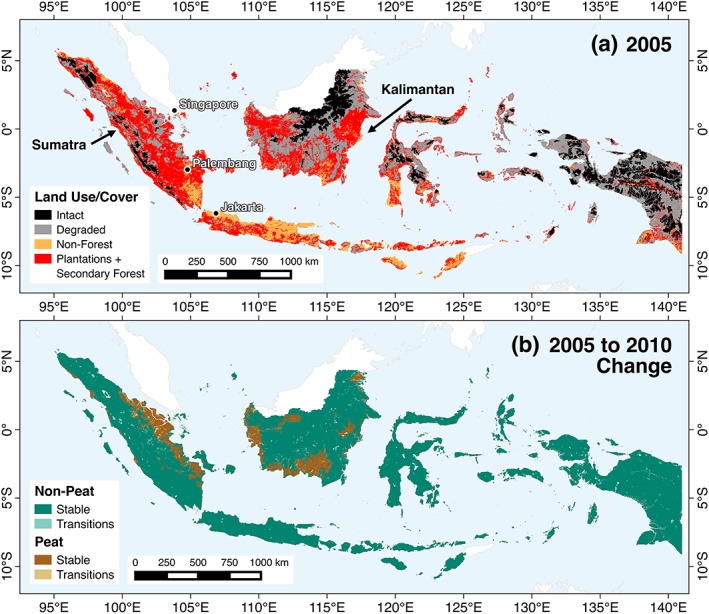
(a) Distribution of 2005 land use and land cover across Indonesia (Hansen et al., 2013; Margono et al., 2014), including intact primary forest, degraded primary forest, nonforested areas, and combined tree plantations and secondary forest. (b) Areas of change from 2005 to 2010 (light shades) and stable areas that did not change (dark shades) in peatland and nonpeatland areas.

Indonesia's fire activity depends on complex interactions between land use and land cover (LULC) change, the extent of burning on carbon‐rich peatlands, and meteorological variability. Indonesia's LULC has rapidly changed over the past few decades (Figure 1). Satellite observations reveal substantial forest loss and degradation across the islands of Sumatra and Kalimantan (Indonesian Borneo) in particular (Margono et al., 2014). Although industrial‐scale plantations account for nearly half of Indonesia's deforestation (Abood et al., 2015), monitoring forest degradation, such as logging, is also important because roughly 80% of degraded forests are eventually cleared (Miettinen et al., 2012). In addition, the magnitude of fire emissions depends on burning in fuel‐rich peatlands. Peatlands are naturally protected from fire by a high water table but become highly flammable following drainage and degradation (Wösten et al., 2008). Nearly 80% of tropical peatlands are located in Indonesia (Page et al., 2011).

Fire activity also varies with meteorology. Higher fire activity typically occurs during droughts associated with El Niño (van der Werf et al., 2008), but recent fire events in non‐El Niño years indicate that localized, intense fires are associated with other meteorological mechanisms (Gaveau et al., 2014; Koplitz et al., 2018). There is a nonlinear sensitivity of fires to drought conditions, particularly below 4‐mm/day precipitation (Field et al., 2016), and anthropogenic influences have increased the susceptibility of the landscape to fires during droughts (Field et al., 2009). Although undisturbed forests rarely burn, large inadvertent fire events (*escaped fires*) associated with combined anthropogenic and drought influences have been observed since the 1960s in Sumatra and 1980s in Kalimantan (Field et al., 2009).

Fires emit organic and black carbon (OC and BC) aerosols (in addition to trace gases) that are the primary components of to smoke‐related surface particulate matter concentrations (Koplitz et al., 2016). Although there are a limited number of epidemiological observational studies focused on health outcomes from fire pollution in equatorial Asia (Ramakreshnan et al., 2018), at a global scale, exposure to fine particulate matter (particles with diameter <2.5 μm, PM_2.5_) from fires is associated with all‐cause mortality, respiratory symptoms, and cardiovascular outcomes (Reid et al., 2016), including serious health impacts for children (Rees, 2016). Public health impacts of fires depend on the magnitude of emissions, atmospheric transport patterns, spatial proximity to population centers, and underlying population characteristics. Fire activity and consequent health impacts vary with meteorological conditions, with a roughly sevenfold increase in estimated mortality observed between opposite phases of the El Niño‐Southern Oscillation (Johnston et al., 2012; Marlier et al., 2013). Atmospheric modeling studies suggest that exceedances above World Health Organization air quality guidelines across the region are largely due to the contribution of Indonesian fires (Marlier et al., 2013). Singapore, for example, is repeatedly affected by fire emissions from peatlands in Sumatra and, to a lesser extent, in Kalimantan (Kim et al., 2015; Marlier, DeFries, Kim, Gaveau, et al., 2015; Reddington et al., 2014).

Potential policy interventions to reduce fires include the Indonesian government's moratorium on granting new industrial plantation concessions in peatlands or primary forests and programs such as Reducing Emissions from Deforestation and Degradation (REDD+). The Peatland Restoration Agency (Badan Restorasi Gambut, BRG) was recently established following the 2015 haze event. BRG's mission is to restore the hydrology of at least 2 million hectares of damaged peatlands in Sumatra, Kalimantan, and Papua over a 5‐year period (https://brg.go.id). Other policy interventions include supply chain certification schemes, such as the Roundtable on Sustainable Palm Oil. Finally, policies focused on cross‐border health impacts include Singapore's Transboundary Haze Pollution Act, which holds plantation companies liable for proven contributions to transboundary haze (Lee et al., 2016; Tan, 2015).

There is growing consensus on the need for government support of fire‐free land management alternatives such as mechanical clearing or wet peat soil cultivation in order to avoid the public health impacts of fires (Carmenta et al., 2017). In this study, we prioritize locations for reducing future fires in order to reduce cumulative downwind exposure to smoke PM_2.5_ from 2020 to 2030, based on future scenarios of LULC and land management. Our interdisciplinary scientific framework to evaluate the public health implications of potential land use decisions in Indonesia is publicly available through an online decision support tool.

## 2 Materials and Methods

### 2.1 Overview

Our modeling framework consisted of the following steps (Figure S1 in the supporting information): (1) quantify trends in past LULC, (2) estimate relationships between observed fire emissions and past LULC, (3) develop a spatially explicit BAU scenario of future trends in LULC and associated fire emissions (from Step 2), (4) calculate the sensitivities of different receptor locations to the location of fire emissions, (5) quantify future exposure to air pollution from fires, and (6) estimate population‐level future health outcomes. We use the BAU future scenario as a baseline case against which to compare multiple spatially explicit fire reduction strategies.

### 2.2 Land Use and Land Cover

The LULC classification was based on the Margono et al. (2014) data set for 2005 and 2010, which mapped primary intact forests, primary degraded forests (subject to forest utilization, such as logging), and nonforested areas. We aggregated the original 30‐m resolution to 1‐km resolution. We also made several modifications. First, within the large nonforested areas (67% and 44% of total area in Sumatra and Kalimantan in 2005), we incorporated the Hansen et al. (2013) Global Forest Change (v1.4) data set to further allocate these areas into areas with tree cover (tree plantations or regrowth) and without tree cover (shrubland, agriculture, or cleared areas). Hansen et al. (2013) mapped annual forest change but did not distinguish between primary (intact or degraded) forests, secondary forests, or tree plantations, so by combining with Margono et al. (2014), we could separate an aggregated tree plantation and secondary forest class from other nonforested areas. Second, we separated peatlands, which have a high fire emissions potential, from nonpeatland areas for all LULC types (Ministry of Agriculture, 2015; Figure 1).

We used Dinamica EGO Version 3.0.17 (Soares‐Filho et al., 2009) to simulate future LULC. Dinamica EGO is a spatially explicit model that uses a Bayesian Weights of Evidence approach to calculate the effect of different spatial variables on a given LULC transition and estimate the spatial probabilities of a given transition. Input data sets were static variables for landform type (Margono et al., 2014), soil type (FAO, IIASA, ISRIC, ISSACAS, JRC, 2012), elevation (Jarvis et al., 2008), slope, protected areas (World Resources Institute, 2017a), oil palm, logging, and wood fiber industrial concessions (World Resources Institute, 2017b, 2017c, 2017e), population density (CIESIN, 2016), distance to roads (CIESIN, 2013), distance to rivers (https://hydrosheds.cr.usgs.gov), and distance to oil palm mills (World Resources Institute, 2017d), as well as dynamic variables that were updated at each model time step and represented the distance to each individual LULC class. We checked for multicollinearity of all input data sets for a variance inflation factor <5. Spatial patterns of change were calibrated with observed behavior from 2005 to 2010.

Using the 2005 and 2010 maps as a training period (Figure S2), we simulated a BAU scenario in 5‐year increments from 2010 to 2030 (Figure S3). The BAU scenario assumes that observed 2005–2010 trends continue in the future and is used as our baseline estimate from which we explore the impact of multiple fire reduction strategies. In 2005, 40% of peatlands and 31% of nonpeatlands in Sumatra were covered by intact or degraded primary forests; by 2010 this had declined to 33% and 30%, respectively (Figures 1 and S3). In Kalimantan, intact and degraded forest coverage declined from 49% to 45% in peatlands and 57% to 56% in nonpeatlands from 2005 to 2010; much of the remaining forest is located in mountainous areas that are difficult to access.

### 2.3 Fire Emissions

We downscaled 0.25° × 0.25° emissions estimates from the Global Fire Emissions Database version 4s (GFED4s; van der Werf et al., 2017) with 1‐km^2^ MODerate resolution Imaging Spectroradiometer (MODIS) Collection 6 fire radiative power (FRP) observations from the Aqua and Terra satellites (Giglio et al., 2016). The Collection 6 algorithm retrieves FRP with a radiance‐based approach, which decreases FRP in all but the most intense fires and helps to capture large fires potentially hidden by smoke and clouds (Giglio et al., 2016). As the original 0.25° × 0.25° fire emissions are too coarse, the downscaled 1‐km^2^ emissions are used to estimate the contribution of individual LULC transitions to monthly emissions while also retaining meteorological variability from 2005 to 2009.

To produce future fire emissions estimates for the BAU scenario over 2010–2029, we first calculated scaling factors, or emissions rates, based on observed monthly fire emissions for each LULC transition type over 2005–2009 for each 0.25° grid cell. Our results are likely somewhat conservative as fire emissions in this period were slightly lower than the 20‐year average (1.3 Tg OC + BC for 2005–2009 versus 1.6 Tg OC + BC for 1997–2016 in Indonesia, for example; van der Werf et al., 2017). In addition, the 2006 El Niño was not as severe as other El Niño events such as 1997 or 2015 (Koplitz et al., 2016; Marlier et al., 2013).

We also accounted for grid cells with nonzero GFEDv4s emissions and no FRP observations by distributing emissions according to the ratio of area for each LULC transition type. We then multiplied individual LULC transition types by the associated monthly emissions per unit area. Fires may occur repeatedly at the same location, for example, burning associated with agricultural maintenance activities or in degraded areas that are susceptible to fires during drought conditions. In these cases, we apply the average associated emissions. We also matched the different fire types from our 1‐km^2^ estimates according to GFED4 categories and applied associated emissions factors for OC and BC (Akagi et al., 2011; van der Werf et al., 2017).

### 2.4 GEOS‐Chem Adjoint Model

Following Kim et al. (2015) and Koplitz et al. (2016), we used the adjoint of the GEOS‐Chem chemical transport model v8‐02‐01 (http://www.geos-chem.org; Bey et al., 2001; Henze et al., 2007) to calculate the sensitivities of population‐weighted PM_2.5_ concentrations in three countries (Singapore, Malaysia, and Indonesia) to regional fire emissions and their spatiotemporal distributions (hereafter referred to as adjoint sensitivities). GEOS‐Chem is driven by GEOS‐5 assimilated meteorology from the NASA Global Modeling and Assimilation Office. Our simulations were conducted at 0.5° × 0.67° horizontal resolution over the nested East Asia domain (70°–150°E, 11°S–55°N; Chen et al., 2009). We focused on OC and BC, the primary constituents of smoke aerosol; the GEOS‐Chem OC/BC simulation is described in detail in Wang et al. (2011). A forward simulation for the high fire period of July–November 2006 using modified GFED3 emissions reproduced PM_10_ observations in the Malaysian Peninsula and Borneo with a spatial correlation coefficient *r* = 0.84 (50 sites) and a mean bias of −27% (Kim et al., 2015). Multiplication of the adjoint sensitivities by the emissions enables us to immediately infer the smoke exposure in the receptor regions for any fire emissions scenario, since the relationship between emissions at the source and smoke exposure at the receptor is assumed to be linear.

For this work, we calculated the adjoint sensitivities given meteorological conditions for the years 2005–2009, yielding a set of monthly mean sensitivity maps spanning these five years. While meteorology is linked to droughts and the incidence of fires, this approach also allowed us to capture some of the interannual variation in the meteorological processes, such as winds and precipitation, which affect smoke transport to the receptors. This ensemble of years included the 2006 El Niño, characterized by strong drought conditions in equatorial Asia. By assuming that the future interannual variability in transport is similar to that in 2005–2009, we can then multiply the sequence of 2005–2009 sensitivities by the future BAU emissions scenario, repeating the sequence every 5 years. In this manner, we derive future exposure to surface PM_2.5_ at the receptors from fire emissions. Validation of modeled PM_2.5_ concentrations is described in the supporting information.

### 2.5 Health Impact Modeling

Population data for 2005 was from the UN‐adjusted GPW data set (CIESIN, 2016). Country‐level population age structure was back‐calculated from the Global Burden of Disease (GBD) project to subset the gridded population data set to adults 25 years of age and older (Global Burden of Disease Collaborative Network, 2017). We then used background mortality rates for these countries from the GBD project (Global Burden of Disease Collaborative Network, 2017) to produce a gridded data set with population 25 years of age and older and background mortality estimates. Our population data do not reflect changes over time as this information was not available at the needed spatial resolution. This likely makes our estimates somewhat conservative due to expected population increases and demographic shifts, but holding other factors constant over time, the error is directly proportionate to the change in population within the age group at risk.

To calculate the excess mortality due to exposure to air pollution from fires occurring mainly in the year following exposure, we used the model‐calculated contribution of fire emissions to PM_2.5_. To represent the relationship between PM_2.5_ exposure and mortality risk in adults, we used a function with a slope of 1.03% increase in annual all‐cause mortality per 1‐μg/m^3^ increase in annual average PM_2.5_ concentrations (95% CI: 0.97–1.11%), from Vodonos et al. (2018), a 53‐study meta‐analysis of mortality risk and long‐term PM_2.5_ exposures. This study used a multivariate linear random effects model and fit a nonlinear parametric function to estimate the slope of the relationship between PM_2.5_ exposure and mortality risk. While our model framework does require the use of a linear function, this function represents a linear approximation of an underlying nonlinear concentration response function that best fits the modeled exposures in this region. For each scenario, the population‐weighted PM_2.5_ concentrations were matched to this curve to find the percentage increase in mortality risk, and then this percentage increase in mortality risk was multiplied by background mortality rate and the total adult population for each receptor country. This method yielded the PM_2.5_ attributable mortality with and without fires, while incorporating nonlinearity at high concentrations. To find the mortality attributable to the fires, we calculated the difference between the two PM_2.5_ estimates.

To calculate the health burden in children, we used a method similar that used for adults. We used total population data from GPW (CIESIN, 2016) and back‐calculated the proportion of children that were under 5 years of age from the GBD (Global Burden of Disease Collaborative Network, 2017). To calculate excess deaths in children under 5, we applied results of a meta‐analysis showing an association between PM_2.5_ exposure and acute lower respiratory infection (ALRI) in children under 5 (Mehta et al., 2011). This analysis indicated a 12% increase in risk of ALRI (95% CI: 3–30%) per 10‐μg/m^3^ increase in annual average ambient PM_2.5_ exposure. We produced a concentration‐response function with a linear 1.2% increase in mortality per 1‐μg/m^3^ increase in annual average ambient PM_2.5_ between 0 and 50 μg/m^3^, and log‐linear at exposure levels above 50 μg/m^3^, consistent with the concentration response function (CRF) from the GBD at annual ambient air pollution levels ≥50 μg/m^3^.

### 2.6 Decision‐Support Tool

The steps above were integrated into an online software tool to support land management decision‐making. The tool quantifies the impact of blocking fire activity in targeted areas—such as peatlands, industrial concessions, protected areas, and planned restoration activities by BRG—on air quality and public health impacts. The online tool also incorporates the flexibility for the user to choose from past fire emissions years (2005 to present) and future fire emissions (until 2029), variable meteorology (2005 to 2009), and receptor regions (Indonesia, Malaysia, and Singapore). Users can also design custom scenarios by exploring fire reduction strategies in individual provinces.

## 3 Results

### 3.1 Development of Future Business‐As‐Usual Scenario

**Figure 2:**
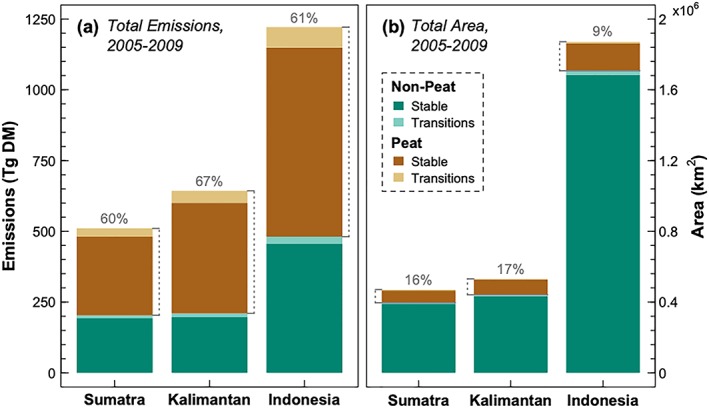
(a) Total emissions estimates (Tg DM) and (b) area (km^2^) for Sumatra, Kalimantan, and all of Indonesia, over 2005–2009. Emissions and area are proportioned into peatlands and nonpeatlands that were stable over the time period or transitioned to a new land use or land cover category. Percentages above the stacked bars represent the contribution of peatlands (stable and transitions) to total emissions or area and indicate the disproportionate influence of peatlands on total emissions.

We first estimated monthly observed emissions for 2005–2010, matching the temporal (5 years) and spatial (1‐km^2^) resolution of the LULC maps (Figure 2). When aggregated to the island or country scale, the largest proportion of emissions contributions was from stable (nontransitioning) LULC classes, especially the combined tree plantations and secondary forest LULC class, followed by nonforested areas, rather than from direct deforestation (Figure S4). Fires in peatlands contributed more to total emissions despite having relatively small total areas, for both aggregated (Figure 2) and individual LULC classes (Figure S4). Finally, most emissions occurred during the months of July to October, and the highest fire years were during 2006 and 2009 El Niño conditions, although Sumatra exhibits a small spring burning season as well (Figure S5).

Our BAU scenario of 2010–2030 LULC projected declines in intact forests and an expansion of the tree plantation and secondary forest class, as well as nonforest (Figure S3). In Sumatra, total forest cover declines from 31% to 24% from 2010 to 2030 and forest cover within peatlands declines from 33% to 8%. BAU trends in Kalimantan predict a decline in intact forest cover from 54% to 49% and 45% to 28% in peatland intact forests. Much of this forest clearance on peatlands is due to expansion of both nonforested areas (13 to 29%) and plantations and secondary forests (54 to 62%) in Sumatra, and nonforested areas in Kalimantan (11 to 27%).

For the analysis of fire emissions and health outcomes associated with the BAU LULC scenario, we focused on the upcoming decade from January 2020 to December 2029. The cumulative emissions over this 10‐year period associated with BAU are 12.7‐Tg OC + BC (Table 1). Across the three receptor regions, this produces July to October PM_2.5_ average population‐weighted exposures of 6.6 μg/m^3^ in Indonesia, 5.5 μg/m^3^ in Malaysia, and 6.0 μg/m^3^ in Singapore and an average of 36,000 excess adult all‐cause deaths every year for the 2020–2029 period. Of these deaths, 92% occur in Indonesia, 7% in Malaysia, and 1% in Singapore. Total regional mortality varies largely with meteorological conditions, ranging from <100 to 80,000 annual deaths depending on the year. This exposure is also associated with 1,100 deaths per year for children under the age of 5 due to ALRI, with 99% of cases in Indonesia (Table S1). While we have projected for the decade from 2020 to 2030, sociodemographic trends suggest that these numbers are likely to be conservative estimates of health effects for the ensuing several decades.

**Table 1:** Cumulative July–October Indonesian fire emissions (Tg OC + BC), average July–October smoke exposure (μg/m^3^ PM_2.5_), and estimated annual average future mortality for Indonesia, Malaysia, and Singapore, from January 2020 to December 2029

Scenario		**Jul–Oct total emissions** (Tg OC + BC)	**Jul–Oct mean smoke exposure** (μg/m^3^)			Annual adult all‐cause mortality		
			Indonesia	Malaysia	Singapore	Indonesia	Malaysia	Singapore
BAU		12.7	6.6	5.5	6	33,000 (31,000–36,000)	2,400 (2,200–2,600)	360 (340–380)
*Remove Fires from:*	*Peatlands*	4.4	2.6	1.6	1.9	12,000 (11,000–13,000)	630 (590–680)	110 (100–110)
	*Concessions*	7.7	4.1	2.2	3.2	19,000 (18,000–20,000)	900 (850–980)	180 (170–200)
	*Conservation areas*	9.6	5.5	5	5.1	29,000 (27,000–31,000)	2,100 (2,000–2,300)	300 (290–330)
	*BRG sites*	7.7	4.1	4.2	3.2	22,000 (21,000–24,000)	1,800 (1,700–2,000)	190 (180–210)

*Note*. First row provides estimates for Business‐As‐Usual (BAU) scenario, and the remaining rows give reductions in emissions, exposure, and health impacts associated with blocking fire emissions in peatlands, industrial concessions, conservation areas, and BRG sites. Ranges in mortality reflect uncertainties in the concentration response function.

### 3.2 Fire Reduction Strategies

**Figure 3:**
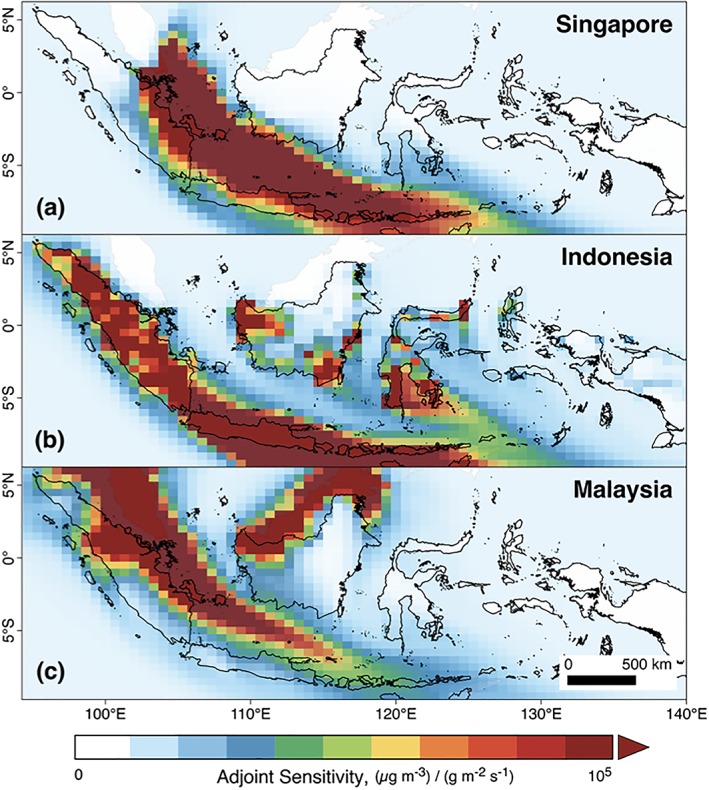
GEOS‐Chem adjoint sensitivities [(μg/m^3^) / (g/m^2^/s)] of the three population‐weighted receptor regions (a Singapore, b Indonesia, and c Malaysia) to the contribution of particulate matter emissions in each grid cell. These examples are for July to October of the 2006 meteorological year. We note that there is an error in the units presented in similar plots of adjoint sensitivities in Kim et al. (2015); Figure 5) and Koplitz et al. (2016); Figure S3).

**Figure 4:**
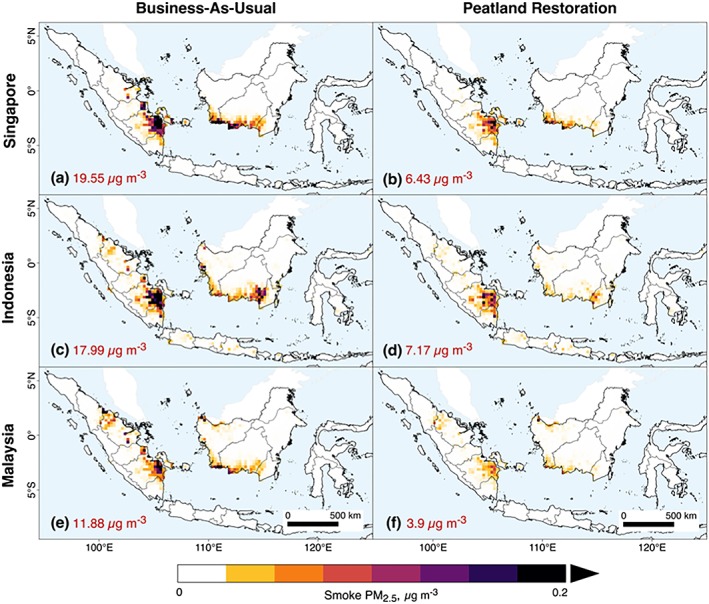
Contribution of PM_2.5_ (μg/m^3^) from fires in individual grid cells to three population‐weighted receptor regions: Indonesia, Malaysia, and Singapore. Example shows how the contribution shifts under the (a, c, and e) Business‐As‐Usual scenario, compared with (c, d, and f) protecting peatlands from fires. These examples show the 2006 meteorological year and 2020 emissions. Mean July–October smoke PM_2.5_ exposure is shown inset.

Different land management strategies can alter the spatial location and magnitude of fire emissions. The adjoint modeling framework applied here examines where fire activity has a disproportionate impact on air quality on selected receptors downwind (Figure 3). Blocking fires in all peatland areas would reduce July to October OC + BC emissions by 65% relative to BAU and average smoke PM_2.5_ exposure by 61% in Indonesia, 71% in Malaysia, and 68% in Singapore (Figure 4). The reduction in average annual adult all‐cause mortality is broadly similar, reducing deaths by 65% in Indonesia, 73% in Malaysia, and 70% in Singapore, relative to BAU (Table 1).

We explored the impact of blocking fires in all industrial oil palm, wood pulp, and logging concessions, which are examples of the options available in the online decision support tool. Blocking such fires reduced July to October OC + BC emissions by 39% and average smoke PM_2.5_ exposures by 38% in Indonesia, 60% in Malaysia, and 47% in Singapore. We also blocked fires in existing conservation areas, which reduced emissions by 24%. Unlike the other scenarios, this scenario reduced PM_2.5_ exposure more in Indonesia (17%) than in Malaysia or Singapore (9% and 15%, respectively), with a similar distribution for associated mortality.

While the scenarios above are focused on average smoke exposures and health responses over the course of a decade, there is also substantial interannual variability. This is largely due to the interplay between meteorological variability and fire activity, such as higher fire activity occurring during a drought year. For our baseline BAU case, our individual annual exposure estimates were calculated by repeating 2005 to 2009 meteorology sequentially in two 5‐year periods for 2020–2029. We find wide variation in smoke exposure in different years. For example, Singapore experiences 20.1 μg/m^3^ in a dry year (such as the 2006 El Niño) but only 0.35 μg/m^3^ in a low‐fire year.

### 3.3 Priority Conservation Areas

Indonesia's BRG has proposed priority peatland restoration locations in eastern Sumatra and southern Kalimantan. These restoration activities aim to restore peatland hydrology to a more natural state. Since the benefit that each individual site could have on reduced fire incidence is highly site specific and variable (Konecny et al., 2016), we explored how our modeling framework could be used to determine which sites would have the most beneficial impact on public health for downwind populations considering a suite of restoration efforts across the a 0.25° grid cell (Table 1). The model prioritizes different restoration efforts for reducing PM_2.5_ concentrations, selecting sites in eastern Sumatra for all receptors, but with a more southern Sumatra focus for the Indonesia receptor. An example of the online tool output is shown in Figure S6. On average, blocking fires in BRG sites would reduce adult mortality by 34% in Indonesia, 22% in Malaysia, and 46% in Singapore. The tool also calculates the top five priority locations of all BRG sites that are most beneficial to public health in terms of reducing PM_2.5_ concentrations for the receptor of interest.

## 4 Discussion

We present a novel approach, based on integration of land use science, atmospheric modeling, and public health, for evaluating the implications of different land management scenarios in Indonesia on regional public health outcomes over the coming decade. This end‐to‐end framework is also relevant to other fire‐prone regions around the world. We found that the effectiveness of different strategies for improving public health outcomes depends on the spatial relationship between the location of fires and population centers and the atmospheric transport patterns that carry emissions from regions experiencing fire to population centers. Science‐based decisions about allocation of scarce restoration and fire reduction strategies require information that is not necessarily intuitive. The online software tool allows decision‐makers to readily assess the public health outcomes associated with land use decisions.

We used a BAU case to explore how alternative land management scenarios could reduce fire emissions, pollution exposure, and public health outcomes in adults and children. Protecting peatlands from fire would reduce July to October total fire emissions by 65% and average smoke exposure by 61% in Indonesia, 71% in Malaysia, and 68% in Singapore. Overall, we calculate that peatland protection could prevent on average 22,000, 1,700, and 250 average excess deaths per year in Indonesia, Malaysia, and Singapore, respectively, into the foreseeable future. If all BRG sites in peatlands are restored, the estimated benefit would be reductions of 11,000, 520, and 160 average deaths per year, respectively.

There are several limitations to this work, but we are primarily focused on the relative changes between a BAU scenario and other fire reduction strategies, an approach which likely helps to reduce the impact of these uncertainties. First, we used LULC maps from 2005 and 2010, as these were based on a consistent methodology to analyze changes over time; newer LULC maps are not always compatible because of changing data sets and methodology (Miettinen et al., 2016). Second, the GFED4s fire emissions inventory and MODIS fire detections may miss fires during peak burning times, which would make our estimates conservative. GFED4s currently uses MODIS C5.1 MCD64A1 burned area (van der Werf et al., 2017), which underestimates the contribution from small fires relative to C6 (Giglio, 2016) and therefore implies lower‐than‐expected fire emissions. There are also improvements in previously unclassified grid cells and omission error. Third, emissions factors of OC and BC from tropical peatland burning are uncertain. Information from new field campaigns suggests that our results may be conservative (Roulston et al., 2018; Wooster et al., 2018). Fourth, there is uncertainty in the health impacts, with our estimates based on studies in the U.S. and Europe. As these estimates are based on epidemiology done on developed countries, they reflect emissions sources, particle constituents, and size distributions, and health care infrastructure of developed countries. Further, these health impacts do not consider the likelihood of increased baseline risk of cardiovascular disease caused by future demographic shifts. Our approach is standard for air pollution health impact assessments, since doing primary epidemiology would involve recruiting a cohort, collecting data on air pollution exposures and relevant confounders, and following them over long periods of time. Unless the entire population is being followed individually and all appropriate confounders are controlled for, an epidemiological approach will not provide a full accounting of impact. Population‐level data are not sufficient since it cannot account for confounders and other population‐level changes. Incorporating newly available data on health outcomes (as well as other input data sets) is a future extension of this work. Finally, although climate changes could alter relationships between fire and atmospheric transport, we do not explore these relationships in the online tool as we are focused on near‐term changes. The influence of climate is an area of future work.

The online tool is publicly available at https://smokepolicytool.users.earthengine.app/view/smoke-policy-tool. While we focused on the maximum benefits of blocking fires in peatlands, industrial concessions, and protected areas, users can also define their own fire reduction strategies in individual provinces and evaluate the benefits for air quality and public health outcomes. For example, we used the tool to rank proposed peatland conservation sites from BRG.

## 5 Conclusion

In this study, we present a novel approach that integrates information on the drivers of fire emissions in Indonesia, the transport of smoke to downwind regional population centers, and the resulting population exposure to air pollution. As a result, this approach quantifies health impacts of different land management scenarios today and into the future. Using this framework, we evaluate the health impact of future fires under different land management scenarios and find that a BAU scenario is likely to lead to an average of 36,000 excess deaths annually into the foreseeable future. However, approximately 66% of this excess mortality could be averted through aggressive peatland restoration efforts. We anticipate that similar integrated approaches might be effective in developing optimal policy approaches to reducing health impacts from biomass burning in other parts of the world.

## Conflict of Interest

The authors declare no conflicts of interest relevant to this study.

## Supporting information



Supporting Information S1
