# Supplementary material for: Fires, Smoke Exposure, and Public Health: An Integrative Framework to Maximize Health Benefits From Peatland Restoration
Source: Geohealth. 2019 Jul 24;3(7):178–89. doi: 10.1029/2019GH000191 (PMC7007093; doi:10.1029/2019GH000191)
Supplement: Supplementary file 1 — Supporting Information S1 [file GH2-3-178-s001.pdf]

GeoHealth

Supporting Information for

Fires, Smoke Exposure, and Public Health: An Integrative Framework to Maximize Health Benefits from Peatland Restoration

Miriam E. Marlier[1,2], Tianjia Liu[3], Karen Yu[4], Jonathan J. Buonocore[5], Shannon N. Koplitz[3], Ruth S. DeFries[2], Loretta J. Mickley[4], Daniel J. Jacob[3,4], Joel Schwartz[6], Budi S. Wardhana[7], & Samuel S. Myers[6,8*]

1. The RAND Corporation, Santa Monica, CA
2. Department of Ecology, Evolution, and Environmental Biology, Columbia University, New York, NY
3. Department of Earth and Planetary Sciences, Harvard University, Cambridge, MA
4. School of Engineering and Applied Sciences, Harvard University, Cambridge, MA
5. Center for Climate, Health, and the Global Environment, Harvard T.H. Chan School of Public Health, Harvard University, Boston, MA
6. Harvard T.H. Chan School of Public Health, Harvard University, Boston, MA
7. Badan Restorasi Gambut, Jakarta, Indonesia
8. Harvard University Center for the Environment, Harvard University, Cambridge, MA

* Corresponding author: smyers@hsph.harvard.edu

Contents of this file

Text S1
Figures S1 to S7
Table S1

Introduction

This supporting information provides additional information on the validation of the GEOS-Chem adjoint model with observational data from Singapore, the emissions modeling framework, and additional health impact calculations for children.

Text S1.

Validation of Adjoint Model with GFEDv4s Emissions

Methods. We used two sources of observational data to compare with our modeled fine particulate matter ($PM_{2.5}$) concentrations. First, we reconstructed monthly 2005-2009 $PM_{2.5}$ based on data from Singapore's National Environment Agency (NEA) and the Global Summary of the Day (GSOD) at the Singapore Changi Airport. Since NEA $PM_{2.5}$ is only available from 2014-2016, we also used daily pollutant standards index (PSI) observations to extend the $PM_{2.5}$ time series to 2010. We then used GSOD meteorological variables (visibility, air temperature, wind speed, and rainfall observations) to model monthly NEA $PM_{2.5}$ from 2010-2016 and reconstruct monthly $PM_{2.5}$ for the 2005-2009 validation period. Second, we used historical $PM_{2.5}$ from news reports. Due to bias from the sparse reporting of $PM_{2.5}$ on highly polluted days, we accounted for missing days in the historical PSI-converted $PM_{2.5}$ by assuming that days with no observations have baseline $PM_{2.5}$ values and weighting the values to obtain monthly $PM_{2.5}$. Baseline $PM_{2.5}$ (13.77 μg m^{-3}) was calculated as the median of $PM_{2.5}$ in January-June and November-December, or non-fire season months. This baseline concentration is similar to what was found by Koplitz et al. (2016). We subtracted baseline $PM_{2.5}$ from the reconstructed $PM_{2.5}$ time series modeled from GSOD meteorological variables and based on historical PSI to infer smoke $PM_{2.5}$. We set months with negative smoke $PM_{2.5}$ to 0 μg m^{-3} to reflect the lower bound of $PM_{2.5}$.

Visibility was the most useful independent variable for reconstructing $PM_{2.5}$. However, since there was an unexplained drop in maximum visibility in mid-2013, we adjusted visibility values after mid-2013 according to the difference in the 95th percentile of visibility before and after May 2013.

Results. Figure S7 shows the results from the validation. The historical PSI-converted $PM_{2.5}$ is shown in blue triangles, the reconstructed $PM_{2.5}$ (daily and monthly averages) is shown in black lines, and monthly modeled $PM_{2.5}$ (from the GFED inventory) is shown in red circles. Since the historical PSI data was taken from news reports, data availability is sparse, but it matches well (r = 0.94) with the reconstructed monthly dataset after accounting for days with no PSI observations. Using monthly mean data, the adjusted r^2 = 0.94, r = 0.97 for reconstructed and observed $PM_{2.5}$.

The modeled smoke $PM_{2.5}$ correlates moderately well with reconstructed (r = 0.69) and historical PSI-converted smoke $PM_{2.5}$ (r = 0.64). While the adjoint model with GFEDv4s emissions accurately captures high smoke $PM_{2.5}$ in October 2006, we see large positive biases in modeled relative to observed $PM_{2.5}$ in September 2006 (+ 31.2 to 31.8) and September 2009 (+ 11.5 to 19). One explanation for this result may be uncertainty in the GFEDv4s experimental small fires boost and burned area date from MCD64A1, which could lead to incorrect allocation of monthly GFED emissions (2006/09-10). High cloud cover over Indonesia results in inconsistent availability of surface reflectance scenes for burned area estimation and less opportunities to observe active fires. Discrepancies in active fires and burned area may also limit the performance of the small fires boost, particularly for pixels with only active fire observations (Zhang et al., 2018). A secondary factor is the low temporal variability in adjoint sensitivities; if daily variability in meteorology (i.e. wind strength and direction) was large over the course of the month, the sensitivity of the Singapore receptor to downwind fire emissions may have also been highly variable.

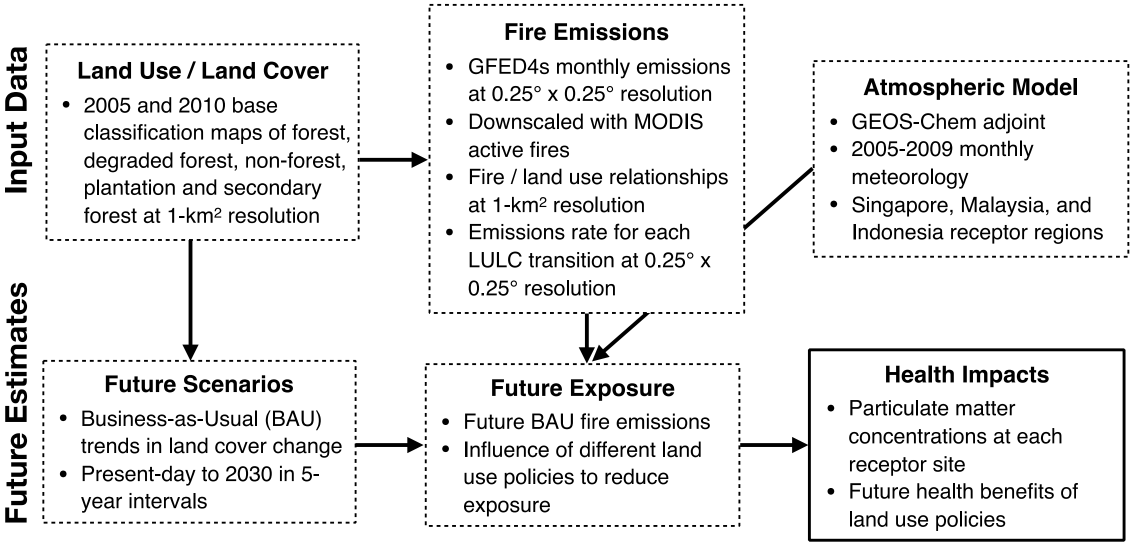

Figure S1. Diagram of input data sources (top row) and steps to produce future estimates (bottom row).

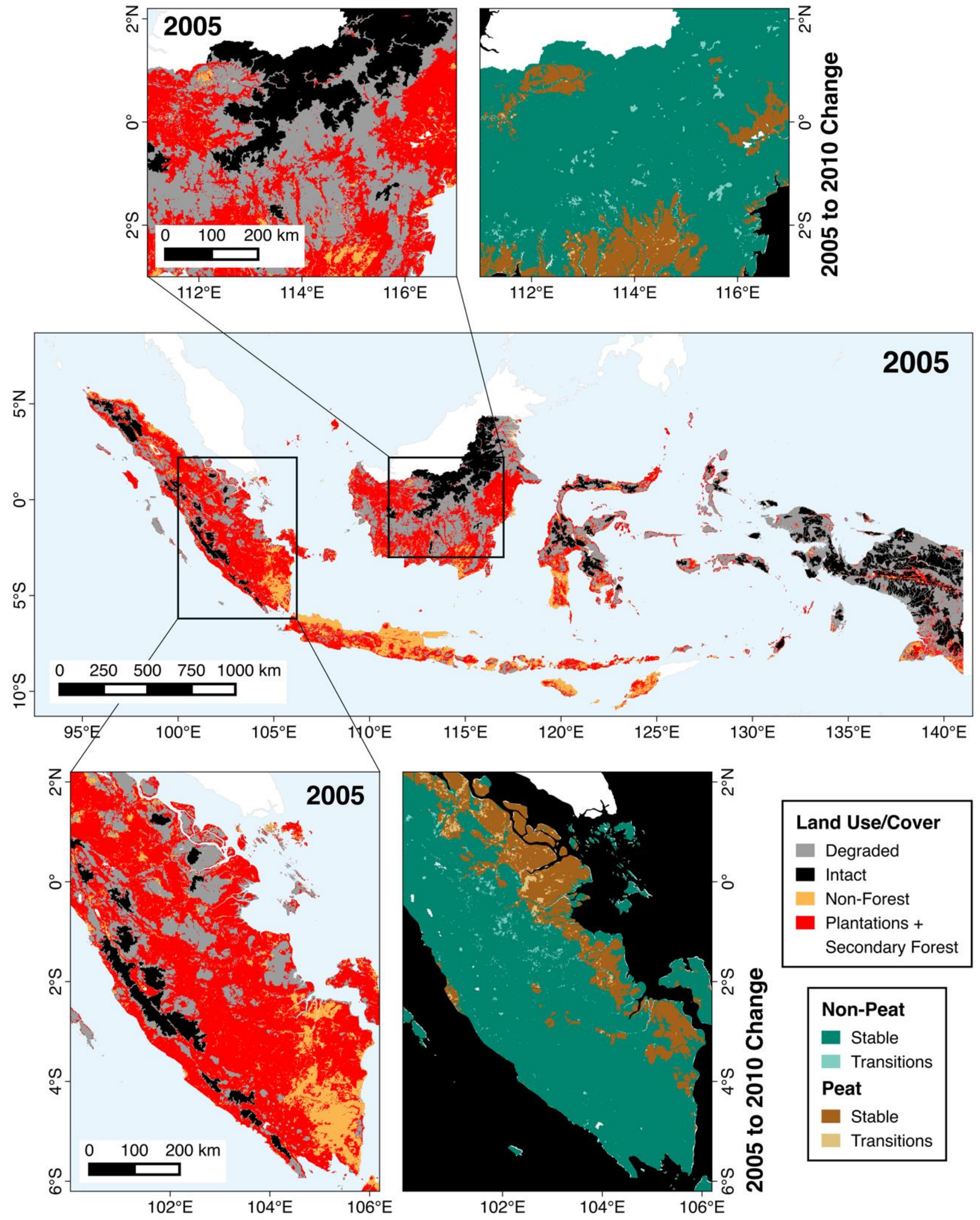

Figure S2. Future area totals for different types of land cover and land use for the Business-As-Usual (BAU) scenario from 2010-2030, every five years. Results are given for Sumatra and Kalimantan, as well as all of Indonesia. Non-peatland areas are given in lighter shades for each category. Intact and degraded refer to natural forest types.

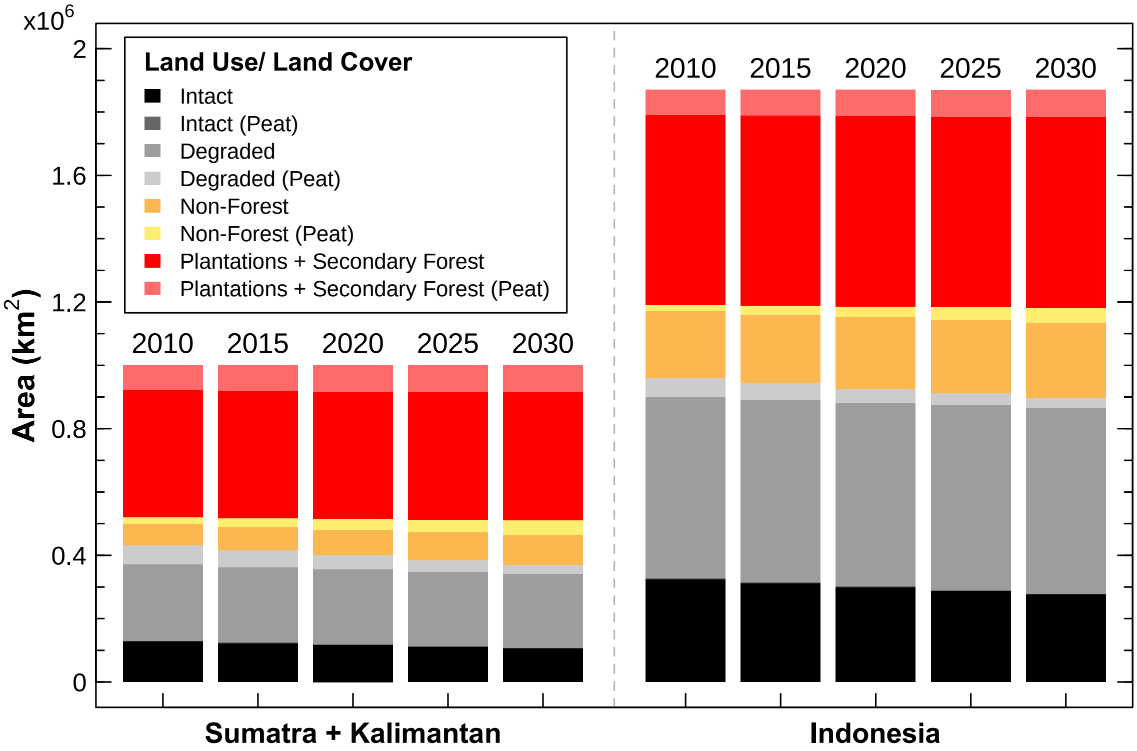

Figure S3. Future area totals for different types of land cover and land use for the Business-As-Usual (BAU) scenario from 2010-2030, every five years. Results are given for Sumatra and Kalimantan, as well as all of Indonesia. Non-peatland areas are given in lighter shades for each category. Intact and degraded refer to natural forest types.

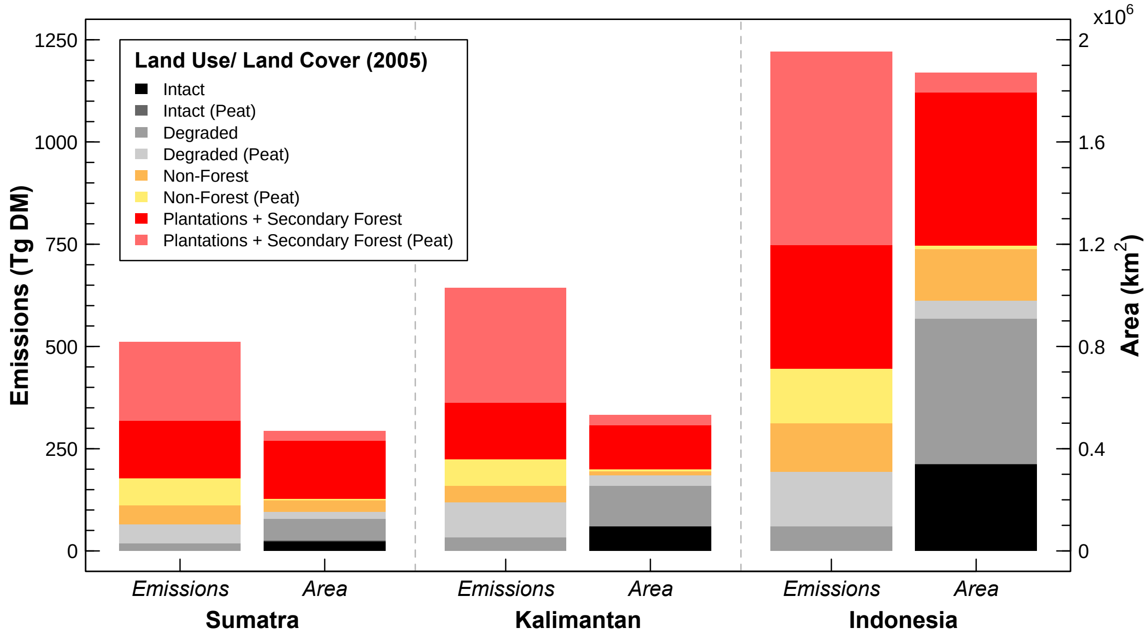

Figure S4. Total emissions and area in 2005 for Sumatra, Kalimantan, and all of Indonesia, for peatland and non-peatland areas in intact forest, degraded forest, non-forest, and plantations with secondary forest.

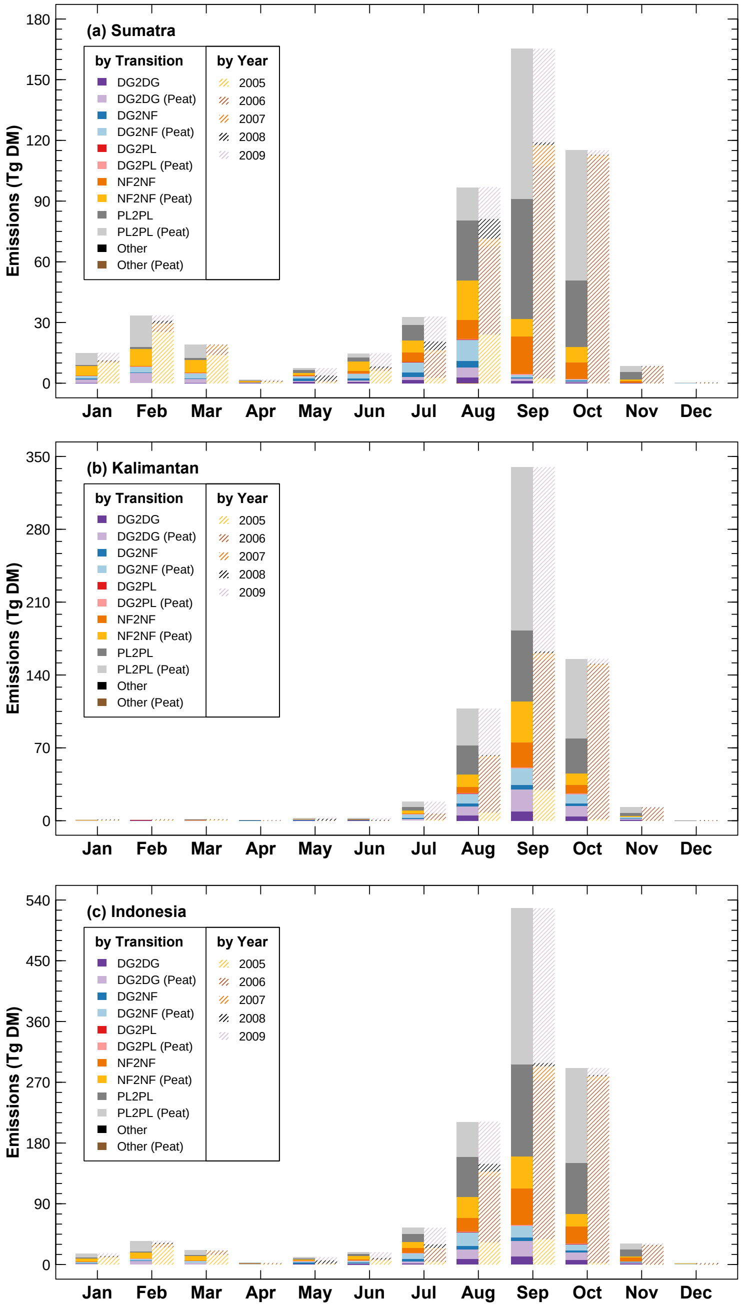

Figure S5. Monthly mean emissions rates for 2005-2009 for (a) Sumatra, (b) Kalimantan, and (c) Indonesia. Codes refer to degraded forest (DG), non-forest (NF), and plantation and secondary forest (PL) on peatland and non-peatland areas.

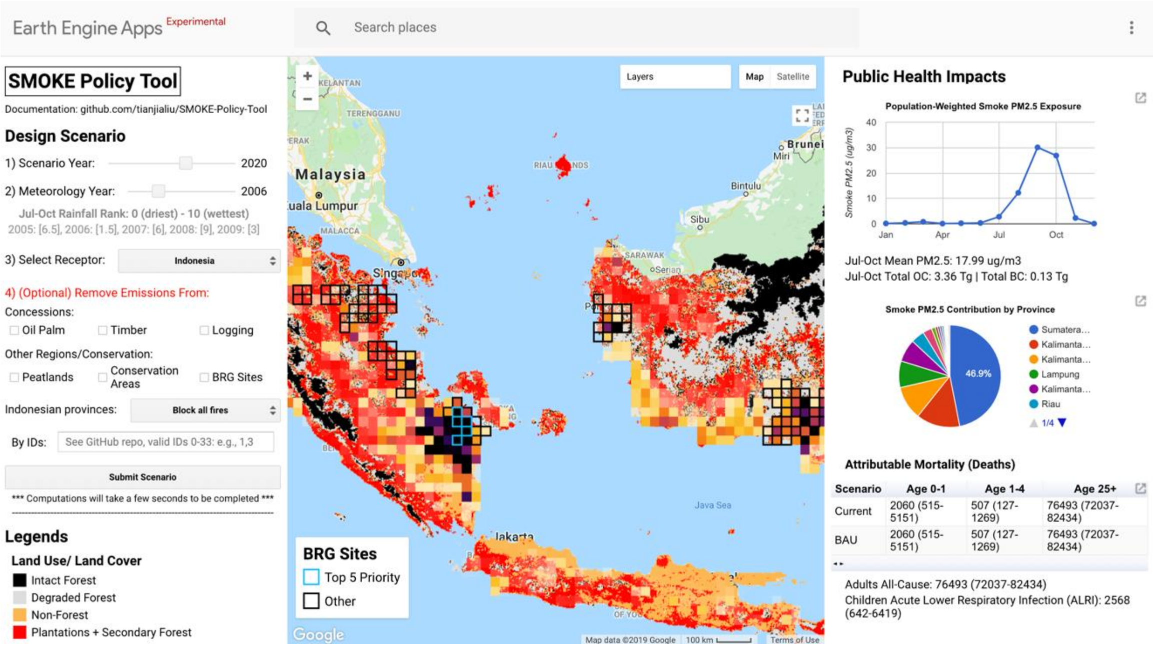

Figure S6. Screenshot of online decision support tool output for blocking fire emissions in grid cells with proposed restoration locations from the Indonesia's Peatland Restoration Agency (BRG). Grid cells with planned restoration activities are in black. Light blue outlines highlight the top five grid cells that contribute to public health impacts for the selected receptor. The background map shows the July-October mean smoke $PM_{2.5}$ exposure (μg/m^3) for Indonesia. Example is shown for the Indonesian receptor for a high fire year (2006 meteorology) and 2020-2025 land use/ land cover transitions.

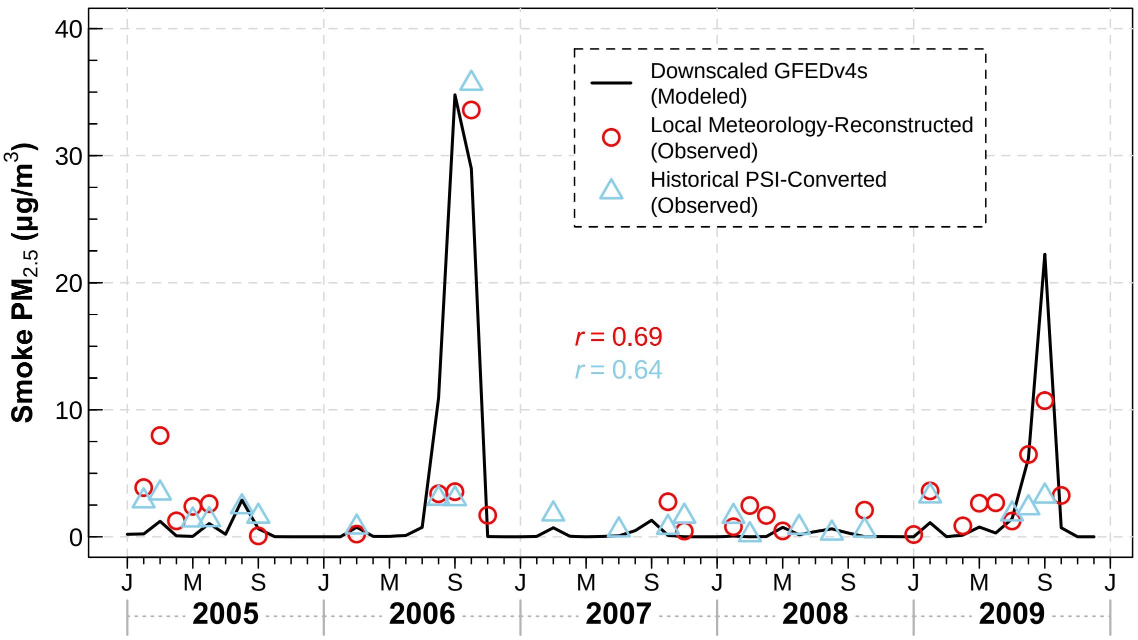

Figure S7. Validation of modeled monthly smoke $PM_{2.5}$ in Singapore, from 2005 to 2009. Historical $PM_{2.5}$ concentrations (blue triangles) are derived from media reports of PSI and weighted by number of observations per month (Alex Pui, personal communication). Reconstructed $PM_{2.5}$ concentrations (red circles) are calculated as a function of observed visibility, air temperature, wind speed, and rainfall from the NOAA Global Summary of the Day (GSOD) dataset for the Singapore Changi airport. We subtract a baseline $PM_{2.5}$ of 13.77 μg m^{-3} from both the historical and reconstructed $PM_{2.5}$ to reflect smoke $PM_{2.5}$; only observed smoke $PM_{2.5}$ observations > 0 are shown. Modeled $PM_{2.5}$ concentrations (black line) are calculated using downscaled GEOS-Chem adjoint sensitivities, GFEDv4s emissions, and information on land use/ land cover transitions. The modeled smoke $PM_{2.5}$ correlates moderately well with reconstructed (r = 0.69) and historical PSI-converted smoke $PM_{2.5}$ (r = 0.64).

Scenario		Jul-Oct Total Emissions (Tg OC+BC)	Jul-Oct Mean Smoke Exposure (µg/m³)			Annual Children ALRI Mortality		
			Indonesia	*Malaysia*	*Singapore*	*Indonesia*	*Malaysia*	*Singapore*
BAU		12.7	6.6	5.5	6	1,122 (280-2,804)	9 (2-23)	0 (0-1)
Remove Fires from:	*Peatlands*	4.4	2.6	1.6	1.9	390 (97-975)	3 (1-6)	0 (0-0)
	Concessions	7.7	4.1	2.2	3.2	637 (159-1,592)	4 (1-9)	0 (0-1)
	Conservation Areas	9.6	5.5	5.0	5.1	958 (239-2394)	8 (2-21)	0 (0-1)
	BRG Sites	7.7	4.1	4.2	3.2	744 (186-1,861)	7 (2-18)	0 (0-1)

Table S1. Cumulative July-October Indonesian fire emissions (Tg OC+BC), average July-October smoke exposure (µg/m^3 $PM_{2.5}$), and estimated annual average future mortality due to acute lower respiratory infection (ALRI) in children, from January 2020-December 2029. First row provides estimates for Business-As-Usual (BAU) land use and land cover trajectories, remaining rows give reductions in emissions and children's health impacts associated with blocking fire emissions in peatlands, industrial concessions, and conservation areas.
